# Mapping susceptibility to air pollution and its association with birth defects: a tool for public health intervention

**DOI:** 10.1093/eurpub/ckaf077

**Published:** 2025-06-11

**Authors:** Carlos Aniceto, Paula Braz, Ausenda Machado, Carlos Matias-Dias

**Affiliations:** Department of Epidemiology, National Institute of Health Doutor Ricardo Jorge, Lisboa, Portugal; Department of Epidemiology, National Institute of Health Doutor Ricardo Jorge, Lisboa, Portugal; Department of Epidemiology, National Institute of Health Doutor Ricardo Jorge, Lisboa, Portugal; Department of Epidemiology, National Institute of Health Doutor Ricardo Jorge, Lisboa, Portugal

## Abstract

Epidemiological studies evaluating the relation of environmental air pollution (AP) and birth defect (BD) are relevant to public health. Some limitations on these studies may derive from multiple factors contributing to the spatial variation of AP. This study aimed to integrate multifactorial AP indicators into an index and explore its application in a case-control study conducted in Portugal between 2016 and 2021. Spatial multicriteria analysis was employed to identify areas susceptible to AP. Variables included: (i) Euclidean distance to industrial units; (ii) kernel estimation of industrial units density; (iii) land occupation; (iv) Euclidean distance to main roads; and (v) areas conductive to radiation fog formation. Variables were classified into high, moderate, and low susceptibility. An AP susceptibility map was generated using the weighted linear combination method, with the analytic hierarchy process assigning weights to the variables. Georeferenced BD cases and controls were overlaid with environmental exposure variables and the AP index. Three AP susceptibility areas were identified: consolidated urban, peri-urban area, and a residential–industrial area. In areas of high susceptibility, 47 cases (29%) and 65 controls (31%) were observed; and in areas of low susceptibility 25 cases (15%) and 21 controls (10%) were observed. The development of the AP susceptibility map has been demonstrated to be a valuable tool for identifying patterns, generating hypotheses regarding the potential environmental exposure of NB to AP agents during pregnancy. When integrated into more complex analyses, these findings may contribute to assess the potential risk factors that play a major role in BD.

## Introduction

Birth defects (BDs) include structural malformations, chromosomal anomalies, and other genetic diseases, and occur during intrauterine life [1]. In developed countries, they are the main cause of perinatal morbidity and mortality, with estimates indicating that around 2%–3% of births have a ‘major’ BD [2]. The etiology of BD is multifactorial, with genetic and environmental factors playing a relevant and often interrelated role [3].

During pregnancy, the use and consumption of substances such as alcohol, drugs, and some medications is known to have adverse effects on the development of the fetus and child [4, 5]. Environmental exposure to some physical and chemical agents has also been associated with the occurrence of BD [6]. Among environmental factors, air pollutants such as particulate matter (PM10 and PM2.5), nitrogen dioxide (NO2), sulfur dioxide (SO2), ozone (O3), and carbon monoxide (CO), are associated with an increased risk of BD [7, 8]. In recent decades, the relationship between air pollution (AP) and BD has been studied, often yielding inconclusive results. This inconsistency arises from challenges in establishing robust associations, primarily due to the limited quantity and quality of available data on exposure [6].

In Portugal, the riverside arc of the south bank of the Tagus River in the Lisbon Metropolitan Area has historically been associated with heavy industrial units. This region still hosts some industrial units in the chemical, fertilizer, and steel sectors. Since the 1990s, industries related to the automobile sector have also emerged. Despite modernization of industrial processes and environmental regulations in accordance with European directives, pollution hotspots persist, requiring ongoing monitoring [9–12].

Therefore, atmospheric emissions from industrial processes or combustion in industrial units may continue to pose risks and can cause impacts on human health. Given the potential health impacts, this issue requires further study, as highlighted by the 2021 review of the World Health Organization's (WHO) air pollutant safety level guidelines [13].

Spatial analysis using geographic information systems (GISs) has become an increasingly utilized methodology in epidemiological studies. The capabilities that GIS offer in analysis and modeling of information, integrating various territorial dimensions, are an asset in studying the health determinants of population, providing essential data for planning actions, implementation, and prevention, as well as for disease control and treatment.

The National Registry of Congenital Anomalies (RENAC) in Portugal is a nosological population-based registry that maintains an epidemiological surveillance system of BD and contributes, among others, to the early warning of new teratogenic exposures. In 2015, an unusual aggregation of cases of newborns (NBs) with anorectal anomalies was detected in the south of Tagus River. It was observed that most of these pregnant women resided in the Setúbal region during their pregnancies, leading to the hypothesis of a geographic cluster.

The study of maternal exposure to teratogenic environmental factors, considering the geographic location of the place of residence during pregnancy and its relationship with the occurrence of BD in NB, is challenging due to the significant variability in exposure assessment [14]. In this context, between 2016 and 2021, in collaboration with the Barreiro Montijo Hospital Center, an epidemiological case-control study was conducted to investigate the association between BD and the environmental exposure of pregnant women based on their area of residence. The aim of the present study was to generate an AP index using a spatial multicriteria analysis (SMCA) methodology and to explore its application in the case-control study [15].

## Methods

### Study design

A SMCA methodology was used to produce an AP index for the study area. This methodology combines and transforms spatial and non-spatial information in a judicious way, based on a scale of values, in a given decision with spatial results [16]. Consequently, a susceptibility map for AP was created, using the weighted linear combination (WLC) and analytic hierarchy process (AHP) methods.

This methodology was applied in an observational, matched case-control epidemiological study conducted between February 2016 and December 2021. The target population for this study comprised NB at the Barreiro Montijo Hospital Center during the study period. All NB with BD (cases) who remained with their mothers during postpartum hospitalization were recruited for the study. For each case, two NB without BD (controls) were selected through systematic sampling, based on the day of birth (Supplementary Table S1).

### Data source, data treatment, and data validation

The data used in the present study are divided into two types: geographic data from various sources and data from the questionnaire survey conducted with the mothers of NBs (Supplementary Table S2).

The validation and consolidation of the survey database were performed by verifying, confirming, and correcting missing values or inconsistencies with the support of the hospital investigation team. Geographic data were collected in GIS vector format and underwent validation to correct associated alphanumeric information and georeferencing errors.

### Study area

A cluster analysis using the Anselin Local Moran's I method was performed to examine the presence of geographic case concentration and define the study area. This method, which employs spatial autocorrelation, identifies local clusters and outliers through the analysis of nearest neighbors [17]. Cases and controls were georeferenced based on the seven-digit postal code corresponding to the mother's residence during pregnancy. A distance of 4 km from each case of NB with anomalies was set as the neighborhood criterion, considering this value as the maximum distance for pollutant emission from an industrial unit [18–20].

### Spatial multicriteria analysis

The SMCA methodology was developed to calculate the AP susceptibility index for the study area. This calculation considered five factors, each contributing to the emission and concentration of air pollutants to varying degrees:

- Distance from polluting economic activities, classified under the Pollutant Emissions and Transfers Registry (PRTR) national protocol as emitting pollutants into the atmosphere, water, and soil, or transferring hazardous waste outside their facilities. In the specialized literature, it is considered that the emission of pollutants by a hazardous activity has a significant impact on the atmosphere within a radius of up to 2000 m [18–22]. Therefore, the distance to PRTR activities was calculated based on the Euclidean distance, which establishes the shortest straight-line distance between two points [23], generating a classified raster output with three levels of susceptibility: high (<2000 m), moderate (2000–4000 m), and low (>4000 m) (Supplementary Table S2).
- Density of polluting economic activities per square kilometer, under the PRTR protocol. It was considered that higher density corresponds to a greater likelihood of increased AP [18, 21, 22]. Consequently, the density of PRTR activities per km^2^ was calculated using Kernel Density Estimation, an interpolation technique and spatial pattern analysis of points that generates a continuous data surface from a set of known points. This method reveals the intensity with which a particular phenomenon is manifested in space [24]. A raster matrix was generated on a continuous scale from 1 to 3 (1 representing the lowest density with low susceptibility and 3 representing the highest density with high susceptibility). The values were reclassified through a linear transformation based on minimum and maximum values (Supplementary Table S2) [25, 26].
- Distance to major road traffic generating routes, including highways, expressways, national roads, external ring roads, and major urban traffic arteries. It was assumed that proximity to these routes correlates with higher levels of pollution [21, 27–29]. To calculate the distance to these routes, roadways were selected based on their functional hierarchy through alphanumeric queries in the OpenStreetMap database [30], as well as from road studies and municipal territorial plans. After this process, the Euclidean distance to the roadways was calculated, defining three levels of susceptibility: high (<100 m), moderate (100–300 m), and low (>300 m) (Supplementary Table S2) [21, 27, 29].
- Land use type and its relationship with the presence of anthropogenic activities that have potential pollutant emissions in a specific area [21, 22, 29]. Accordingly, land use data were classified into three susceptibility categories based on potential pollutant emission capacity: high—industrial areas, roadways, airports, waste treatment facilities, landfills, and dumps; moderate—residential areas, commercial spaces, recreational, and tourism facilities; low—natural, forested, and agricultural areas (Supplementary Table S2) [21, 29].
- Areas conducive to radiation fog formation, susceptible to pollutant accumulation due to the tendency of pollutants to adhere to water particles and the limited dispersion caused by the absence of wind. In the south bank of the Tagus River in the Metropolitan Area of Lisbon, radiation fog is particularly frequent in low-lying regions due to the drainage of cold air along slopes and its concentration in topographically depressed areas [31, 32]. To identify these areas, a fog map published by the Lisbon Metropolitan Area Authority was used. Two levels of susceptibility were defined: high (areas prone to radiation fog formation) and low (areas not prone to radiation fog formation) (Supplementary Table S2) [31].

To perform the SMCA, the emission and pollution concentration factors mentioned above were used as inputs for the susceptibility map. For this purpose, these factors were spatially mapped and combined using the WLC method [15, 16, 33], employing the following equation:


(1) S=Σi wi*xi

In this equation, the variables (*xi*) are weighted by weights (*wi*), according to their importance, and then summed, resulting in a degree of favourability for a specific objective (*S*).

The AHP method, a multiple-criteria decision-making tool widely used in various scientific fields, relies on influence scales and pair-wise comparisons of indicators. This method was employed to weight the variables according to their importance in the emission and concentration of pollution [34–36]. The AHP was conducted in three main stages: establishing the paired comparison matrix for the factors; determining and normalizing the relative importance of each factor; and checking the consistency ratio during the comparison process (Table 1). The relative importance of each factor was determined based on literature, with greater weight assigned to PRTR activities in the production of pollutants, as detailed in Table 1 [18–21, 27, 29, 31].

**Table 1.** AHP pair-wise comparison (intensity of importance) and weights

	Distance to PRTR activities (m)	Density of PRTR activities per km²	Land use type	Distance to major roadways (m)	Areas conducive to radiation fog formation	Weight
Distance to PRTR activities (m)	1	1	5	5	7	0.37
Density of PRTR activities per km²	1	1	5	5	7	0.37
Land use type	1/5	1/5	1	3	5	0.09
Distance to major roadways (m)	1/5	1/5	1/5	1	5	0.13
Areas conducive to radiation fog formation	1/7	1/7	1/5	1/5	1	0.04
					CR	0.09

Legend: 1 = equal importance; 3 = moderate importance; 5 = strong importance; 7 = very strong importance. CR = consistence ratio. Dark grey cells indicate a value of 1, representing comparisons where the same variable is evaluated against itself. Light grey cells highlight the Consistency Ratio (CR) values, which assess the consistency of the pairwise comparisons.

The variables were spatially represented and converted into raster format with 10 m cell size. The data were then normalized and reclassified on a scale from 1 to 3, with 1 indicating low susceptibility, 2 indicating moderate susceptibility, and 3 indicating high susceptibility (Supplementary Table S2). The cell values of the factor maps were multiplied by their respective influence percentages and summed, resulting in a map of AP susceptibility categorized into three classes: high, moderate, and low.

Descriptive and bivariate analyses were performed, and both absolute and relative frequencies were calculated to characterize the cases and controls. Pearson's chi-square test was used to assess statistical significance between the observed values of environmental exposure variables and the cases and controls. To analyse the association between cases and controls, the odds ratio was calculated using Kriging interpolation, a method that generates a continuous surface of values based on a set of known data points [25].

## Results

### Cluster analysis

**Figure 1.**
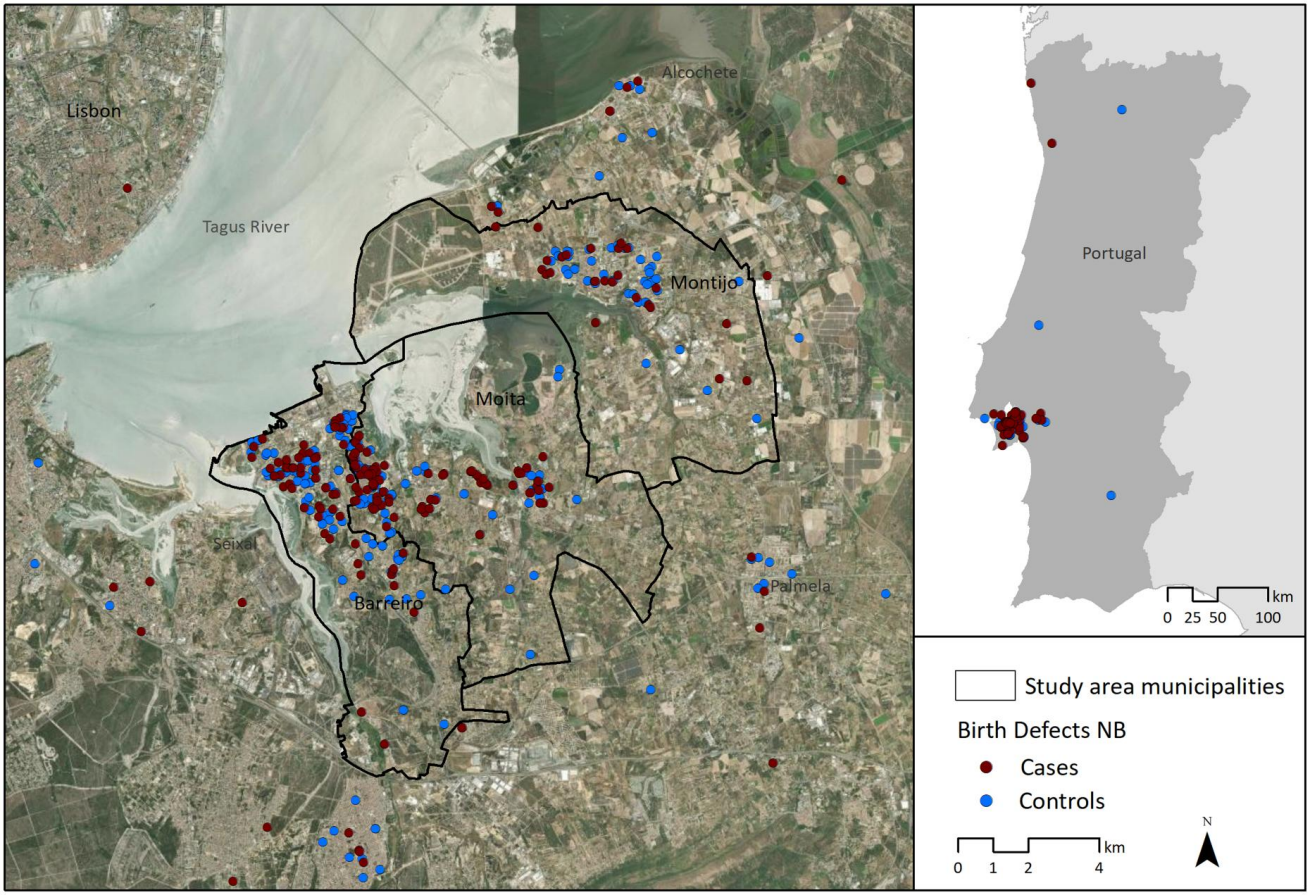
Georeferencing of the mothers' residential area for the cases and controls recruited for the study between 2016 and 2021.

The study area defined for the analysis includes the municipalities of Barreiro, Moita, and Montijo, located in the Lisbon Metropolitan Area. This territory was selected due to its high number of cases and controls, with 164 cases and 209 controls (Supplementary Table S1), which enhances the robustness of the analysis (Fig. 1).

As a result, a spatial cluster of cases (High–High Cluster) was identified in Barreiro, Lavradio, Alto Seixalinho, Baixa da Banheira, and Alhos Vedros (*z*-score >2.25 and <5.76; *P* ≥ .002 and ≤.016) (Supplementary Fig. S1).

### Emission factors and concentration of air pollutants

- Distance to PRTR activities: higher susceptibility to AP was observed in the consolidated urban areas of Barreiro and Baixa da Banheira, attributed to their proximity to an industrial park. Similar patterns are evident in the urban area of Santo André and the peri-urban area of Palhais, influenced by their proximity to a steel industry site. Increased susceptibility is also noted around the automobile industry cluster and a landfill near Palmela, as well as in the region between the southern urban area of Montijo and Alto do Estanqueiro—Jardia, due to the Montijo industrial core (Supplementary Fig. S2).
- Density of PRTR activities per km^2^: the highest density of PRTR activities was found in the consolidated urban areas of Barreiro, Baixa da Banheira, and Santo André, as well as the peri-urban area of Palhais. These areas exhibit the greatest susceptibility to AP (Supplementary Fig. S3).
- Distance to major roadways: the results revealed that the primary road network is significantly denser in the three main urban centers (Barreiro, Moita, and Montijo). This higher density may impact air quality in these areas due to pollutants emitted by road traffic (Supplementary Fig. S4).
- Land use type: the output from the land use analysis demonstrated significant fragmentation of industrial activities within the study area. Although these activities are not categorized as PRTR activities, they have the potential to be sources of pollution (Supplementary Fig. S5).
- Areas conducive to radiation fog formation: radiation fog is quite frequent in the lower Tagus Valley during the autumn and winter months due to the accumulation of cold, humid air from maritime air masses at lower elevations [31]. As result, the riverside areas in the study region were classified as highly susceptible to AP (Supplementary Fig. S6).

### Air pollution susceptibility map

**Figure 2.**
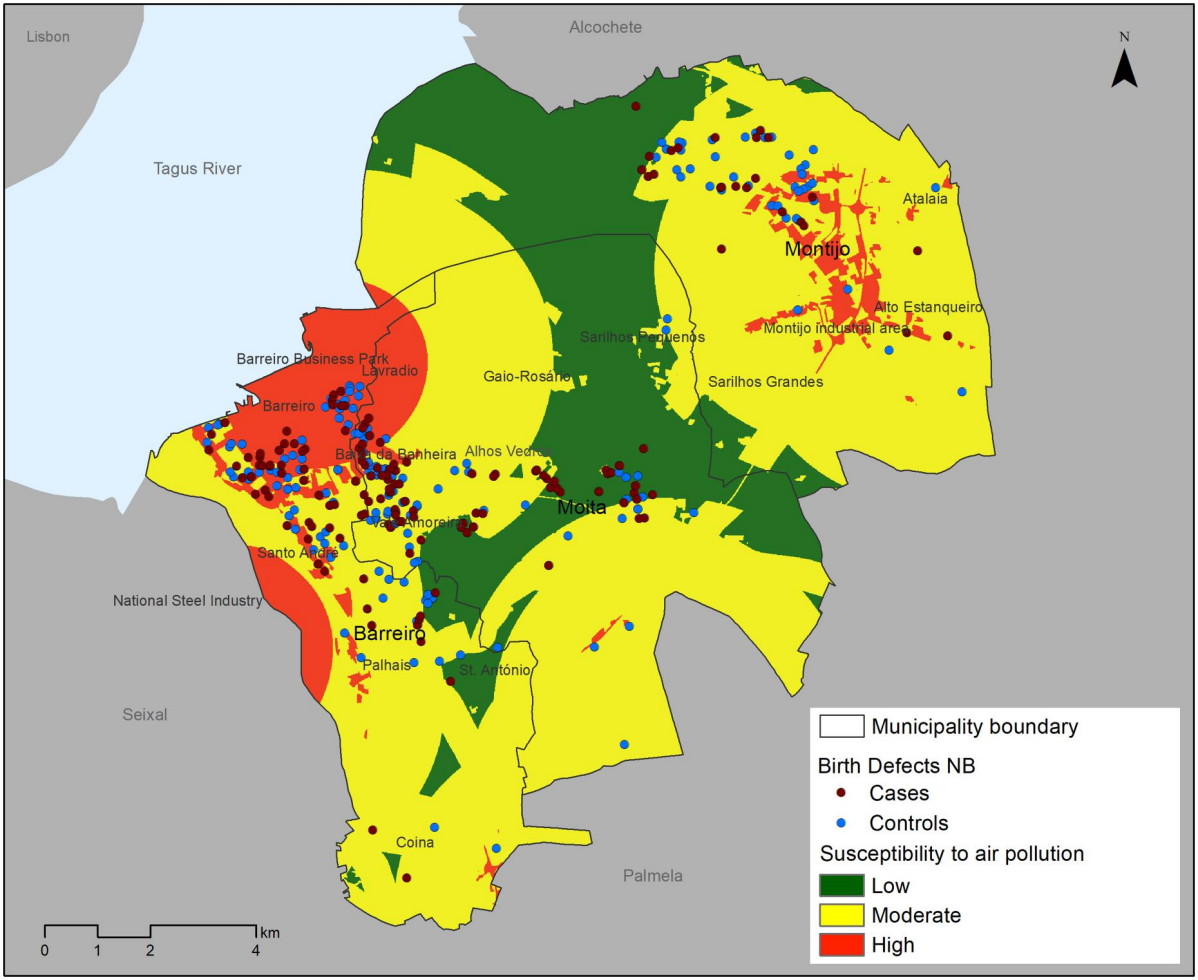
Map of air pollution susceptibility for the residential areas of mothers of cases and controls recruited for the study between 2016 and 2021.

In geographical terms, areas with higher susceptibility to AP were found in the consolidated urban center of Barreiro and Baixa da Banheira, which are densely populated and close to an industrial park and the riverside area; in the peri-urban space of Palhais, situated on the bank of the Tagus River to the east of the steel industry site; and in the southern urban area of Montijo, characterized by numerous economic activities and high population density (Fig. 2). These locations accounted for a significant proportion of cases, comprising 28.6% of the total, compared to 31.1% of the controls. Areas with moderate AP contained the majority of cases (56.1%) and controls (58.8%). According to the results, no significant association was observed between susceptibility to AP and cases of BD (*P* = .31) (Table 2).

**Table 2.** Absolute and relative frequency of cases and controls based on environmental exposure, according to the residential areas of mothers recruited for the study between 2016 and 2021

Environmental exposure	Case		Control		
	*n*	%	*n*	%	*P*
Susceptibility to air pollution					.314
Low	25	15.2	21	10.5	
Moderate	92	56.1	123	58.8	
High	47	28.7	65	31.1	
Distance to PRTR activities					.045
Low	19	11.6	11	5.3	
Moderate	82	50.0	100	47.8	
High	63	38.4	98	46.9	
Density of PRTR activities per km²					.259
Low	43	26.2	41	19.6	
Moderate	95	57.9	137	65.5	
High	26	15.8	31	14.8	
Distance to major roadways					.927
Low	31	18.9	37	17.7	
Moderate	70	42.7	91	44.5	
High	63	38.4	81	37.8	
Land use type					.017
Low	6	3.6	17	8.1	
Moderate	150	91.5	190	90.9	
High	8	4.8	2	1.0	
Areas conducive to radiation fog formation					.922
Low	12	7.3	17	8.1	
High	152	92.7	192	91.9	
Residence municipality					.242
Barreiro	67	31.6	98	36.8	
Moita	73	34.4	68	25.6	
Montijo	29	13.7	47	17.7	
Residence Parish					.247
Barreiro—Barreiro e Lavradio	14	6.6	28	10.6	
Barreiro—Palhais e Coina	4	1.9	4	1.5	
Barreiro—Santo António da Charneca	6	2.8	17	6.4	
Barreiro—Alto do Seixalinho. Santo André e Verderena	36	16.9	35	13.3	
Moita—Moita	14	6.6	14	5.3	
Moita—Baixa da Banheira e Vale da Amoreira	43	20.3	49	18.6	
Moita—Alhos Vedros	22	10.4	16	6.1	
Moita—Gaio-Rosário e Sarilhos Pequenos	^a^	^a^	^a^	^a^	
Mocntijo—Atalaia e Alto Estanqueiro-Jardia	3	1.4	4	1.5	
Montijo—Montijo e Afonsoeiro	21	9.9	38	14.4	
Montijo—Sarilhos Grandes	^a^	^a^	^a^	^a^	

a The criterion of statistical secrecy was applied for absolute frequencies <3.

The analysis of pollution factors revealed a statistically significant association between the cases and the land use type (*P* = .017), with 4.9% of cases and 1.0% of controls observed in areas of high susceptibility. Additionally, a statistically significant association was found between distance to PRTR activities and controls (*P* = .045), with 38.1% of cases and 46.9% of controls located in high susceptibility areas. Analysing the place of residence of the mothers, although statistical significance was not found, a higher proportion of cases (34.4%) compared to controls (25.6%) was observed in the Moita municipality. Within this municipality, a similar pattern was observed in the parishes of Baixa da Banheira and Vale da Amoreira (20.3% versus 18.6%) and in the parish of Alhos Vedros (10.4% versus 6.1%). In the other municipalities studied, a higher proportion of cases compared to controls was noted in the parishes of Alto do Seixalinho, Santo André, and Verderena (16.9% versus 13.3%), as well as in the parish of Moita (6.6% versus 5.3%) (Table 2).

The dichotomy of cases and controls was analysed using the odds ratio calculated through the Kriging interpolation method. This analysis identified a higher proportion of cases relative to controls in the areas of Santo André, Baixa da Banheira, Vale da Amoreira, Alhos Vedros, and Moita (Supplementary Fig. S7).

## Discussion

Our study hypothesized that a geographical cluster of BD could be related to maternal exposure to teratogenic factors, specifically AP. To investigate this, an AP susceptibility map was generated using a SMCA methodology.

As main result, we demonstrated that spatial analysis methodologies and techniques related to the overlay and prediction of information are valuable tools for mapping susceptibility to AP. These methods can significantly contribute to analysing its potential impact on fetal and child health during pregnancy.

The results revealed no association between spaces with higher susceptibility to AP and the place of residence of the mothers of BD cases during pregnancy. However, when analysing the pollution factors individually, a significant association was found with the type of land use. It was also observed that in some parishes and localities, there is a higher proportion of cases compared to controls in areas with high susceptibility to AP.

Most studies on maternal exposure to AP and its relationship with BD in NB focus on analysing the correlation between BD and specific air pollutants or the proximity to particular sources of AP [6, 14, 37]. While SMCA is widely used in various scientific disciplines, including environmental, socioeconomic, and urban planning studies, its application for developing susceptibility indices for AP in epidemiological studies of BD is limited [15, 36, 38].

The SMCA applied in this study utilized the WLC and AHP methods to spatially cross pollution factors, considering their characteristics, manifestation in the territory, and importance in pollutant emission intensity and concentration. The classification and weighting of each factor included in the final model required careful justification, which was based on a bibliographic analysis of the topic under study.

In that way, and considering the studies conducted on pollution sources in the study area, as well as on AP exposure and its association with BD, it was determined that polluting economic activities were the most important source of pollution [6, 9–12]. Additionally, a critical point of this methodology was the establishment of the AP susceptibility class for each factor. The literature suggests that emissions from polluting activities can significantly impact the atmosphere within a radius of up to 2 km from the source. Some authors argue that the impacts may extend up to 4–6 km, although it is generally accepted that beyond this distance, the impacts become minimal. However, it is commonly assumed that the proximity and density of these activities constitute a significant risk factor for human health [18–22]. Regarding PRTR activity density ranges, to the best of our knowledge, no established thresholds in the literature directly correlate their density with pollution levels. Therefore, pollution susceptibility classes were defined based on their density within the study area. It is also well established that the closer the proximity to main routes generating road traffic, the higher the pollution levels. Although some studies define different distances as susceptibility criteria, we considered that the places of residence most susceptible to AP are those located less than 100 m from these roads. For locations more than 300 m away from the roads in question, it was considered that the impact on human health is significantly reduced [21, 27–29]. In the study area, radiation fog occurs notably in autumn and winter [31]. Despite this, we considered that areas conducive to the formation of radiation fog are a factor in pollutant concentration and should be included in the AP index.

Assessing maternal environmental exposure to AP during pregnancy is a challenging process due to the complexity of measuring and spatializing the emission and dispersion of pollutants. The insufficiency of concrete environmental data regarding pollutant emissions was a limitation of this study. Moreover, despite their importance in pollutant dispersion, predominant wind direction and speed were not considered due to insufficient meteorological and satellite data for the study area. Anticyclonic blocking would better indicate pollutant non-dispersion, however, given the data limitations of this small-area study, radiation fog-prone areas were used as a proxy variable. Another limitation of the study arises from potential sample selection bias, as women who chose to terminate their pregnancy due to severe BD in the fetus or who had a stillbirth were not included in the study. Additionally, women residing in the geographical area of the study could have had their children in other hospitals. Finally, there may have been a bias in the classification of cases and controls in terms of their exposure, since exposure was measured at the territory level and not at the individual level.

Despite the challenges epidemiological studies face in establishing causal links between BD and AP, the study of BD can benefit from using a SMCA methodology with its spatial multivariable crossing capacity. This study also enhances the monitoring and surveillance capabilities of RENAC by utilizing the 7-digit postal code, enabling highly detailed analysis down to the street level. Although statistically significant associations between maternal residence and BD were not found, there may be clinical relevance in recognizing that pregnant women residing in specific neighborhoods and areas in Barreiro and Moita have a higher proportion of births with BD. Moreover, it demonstrates the potential of utilizing spatial analysis methodologies in public health and epidemiology, as these methods generate information and build knowledge that uncover important spatial patterns for decision-making purposes. Additionally, an advantage of this index is its applicability to other geographical areas and its use in studying environmental exposure related to specific anomalies.

To better understand the connection between BD and environmental exposure to chemical agents, future studies should focus on analysing the association between BD and records of pollutant emissions into the atmosphere, such as PM10, NO2, and SO2. Additionally, it is important to consider the mothers' places of work and leisure in these analyses.

The creation of a map of susceptibility to AP proved valuable for assessing potential environmental exposure of pregnant women to chemical agents. When integrated into a more comprehensive analysis, these results can contribute to the investigation of possible causal links between BD and environmental exposure. The ability of spatial analysis to associate variables in a judicious way, at a very detailed scale, and create scenarios was demonstrated.

## Supplementary Material

ckaf077_Supplementary_Data

## Data Availability

The data underlying this article will be shared on reasonable request to the corresponding author. Key points A case-control study was conducted to investigate the association between birth defects and maternal environmental exposure during pregnancy. Spatial multicriteria analysis methodology was applied using geographic information system software. Five environmental factors were considered, each contributing to the emission and concentration of air pollutants in varying degrees: distance from polluting economic activities, density of polluting economic activities per square kilometer, distance to major road traffic, land use type and areas conducive to radiation fog formation. A spatial index of air pollution susceptibility was generated. Spatial analysis methodologies can be valuable tools for public health intervention.

## References

[1] World Health Organization. *Congenital Anomalies* [Internet]. 2023. https://www.who.int/health-topics/congenital-anomalies#tab=tab_1

[2] Lanzoni M , Morris J, Garne E et al European monitoring of congenital anomalies. JRC Technical Reports. 2017.

[3] Mendes IC , Jesuino RSA, Pinheiro D da S et al Anomalias congênitas e suas principais causas evitáveis: uma revisão. Rev Méd Minas Gerais 2018;28:1–6. http://www.rmmg.org/artigo/detalhes/2329%0Ahttp://fi-admin.bvsalud.org/document/view/8v8w2

[4] Dyląg KA , Anunziata F, Bandoli G et al Birth defects associated with prenatal alcohol exposure—a review. Children 2023;10:811. https://www.mdpi.com/2227-9067/10/5/811 37238358 10.3390/children10050811 PMC10217313

[5] Vossler DG. Comparative risk of major congenital malformations with 8 different antiepileptic drugs: a prospective cohort study of the EURAP registry. Epilepsy Curr 2019;19:83–5. 30955418 10.1177/1535759719835353 PMC6610403

[6] Baldacci S , Gorini F, Santoro M et al Environmental and individual exposure and the risk of congenital anomalies: a review of recent epidemiological evidence TT—Esposizione ambientale e individuale e rischio di anomalie congenite: una rassegna delle evidenze epidemiologiche recenti. Epidemiol Prev 2018;42:1–34. 10.19191/EP18.3-4.S1.P001.057 30066535

[7] Sun S , Zhang Q, Sui X et al Associations between air pollution exposure and birth defects: a time series analysis. Environ Geochem Health 2021;43:4379–94. 10.1007/s10653-021-00886-2 33864585

[8] Zhang J-Y , Wu Q-J, Huang Y-H et al Association between maternal exposure to ambient PM10 and neural tube defects: a case-control study in Liaoning Province, China. Int J Hyg Environ Health 2020;225:113453. 31986338 10.1016/j.ijheh.2020.113453

[9] CCDR-LVT. Avaliação da qualidade do ar ambiente na região de Lisboa e Vale do Tejo em 2015. 2016.

[10] CCDR-LVT. Avaliação da qualidade do ar ambiente na região de Lisboa e Vale do Tejo em 2021. 2022, 1–59.

[11] Agência Portuguesa do Ambiente. Registo de Emissões e Transferência de Poluentes (PRTR) [Internet]. 2023. https://apambiente.pt/avaliacao-e-gestao-ambiental/registo-de-emissoes-e-transferencia-de-poluentes-prtr

[12] CCDR-LVT. Plano de Melhoria da Qualidade do Ar da Região de Lisboa e Vale do Tejo para os poluentes partículas PM10 e dióxido de azoto nas aglomerações da Área Metropolitana de Lisboa Norte e da Área Metropolitana de Lisboa Sul. 2017. https://www.ccdr-lvt.pt/wp-content/uploads/2022/02/Segundo-Plano-Melhoria-Qar-RLVT-PoluentesParticulas-DioxidoAzoto.pdf

[13] World Health Organization. WHO global air quality guidelines: particulate matter (PM2.5 and PM10), ozone, nitrogen dioxide, sulfur dioxide and carbon monoxide [Internet]. 2021. https://apps.who.int/iris/bitstream/handle/10665/345329/9789240034228-eng.pdf?sequence=1&isAllowed=y 34662007

[14] Huang X , Chen J, Zeng D et al The association between ambient air pollution and birth defects in five major ethnic groups in Liuzhou, China. BMC Pediatr 2021;21:232. 33990187 10.1186/s12887-021-02687-z PMC8120832

[15] Malczewski J , Jankowski P. Emerging trends and research frontiers in spatial multicriteria analysis. Int J Geogr Inf Sci 2020;34:1257–82. https://www.tandfonline.com/doi/full/10.1080/13658816.2020.1712403

[16] Malczewski J. Spatial multicriteria decision making and analysis. In: Thill JC (ed.), Spatial Multicriteria Decision Making and Analysis. London: Routledge, 2019, 11–48. https://www.taylorfrancis.com/books/9780429791529

[17] Anselin L. Local indicators of spatial association—LISA. Geogr Anal 1995;27:93–115. https://onlinelibrary.wiley.com/doi/10.1111/j.1538-4632.1995.tb00338.x

[18] Santos-Sánchez V , Córdoba-Doña JA, García-Pérez J et al Cancer mortality and deprivation in the proximity of polluting industrial facilities in an industrial region of Spain. Int J Environ Res Public Health 2020;17:1860. 32183043 10.3390/ijerph17061860 PMC7142953

[19] Pascal M , Pascal L, Bidondo M-L et al A review of the epidemiological methods used to investigate the health impacts of air pollution around major industrial areas. J Environ Public Health 2013;2013:737926. 23818910 10.1155/2013/737926 PMC3684125

[20] Kihal-Talantikite W , Zmirou-Navier D, Padilla C et al Systematic literature review of reproductive outcome associated with residential proximity to polluted sites. Int J Health Geogr 2017;16:20. 28558782 10.1186/s12942-017-0091-y PMC5450119

[21] Han L , Zhao J, Gao Y et al Spatial distribution characteristics of PM2.5 and PM10 in Xi’an City predicted by land use regression models. Sustain Cities Soc 2020;61:102329. https://linkinghub.elsevier.com/retrieve/pii/S2210670720305503 32834929 10.1016/j.scs.2020.102329 PMC7293537

[22] Li X , Xu Y, Yao X. Effects of industrial agglomeration on haze pollution: a Chinese city-level study. Energy Policy 2021;148:111928. 10.1016/j.enpol.2020.111928

[23] Danielsson PE. Euclidean distance mapping. Comput Graph Image Process 1980;14:227–48. https://linkinghub.elsevier.com/retrieve/pii/0146664X80900544

[24] Shi X , Li M, Hunter O et al Estimation of environmental exposure: interpolation, kernel density estimation or snapshotting. Ann GIS 2019;25:1–8. https://www.tandfonline.com/doi/full/10.1080/19475683.2018.1555188 30687456 10.1080/19475683.2018.1555188 PMC6345173

[25] Smith M , Goodchild M, Longley PA. Geospatial analysis—a comprehensive guide to principles techniques and software tools. 2018. https://www.spatialanalysisonline.com/HTML/

[26] Jenks GF , Caspall FC. Error on choroplethic maps: definition, measurement, reduction. Ann Assoc Am Geogr. 1971;61:217–44. http://www.tandfonline.com/doi/abs/10.1111/j.1467-8306.1971.tb00779.x

[27] Habermann M , Medeiros APP, Gouveia N. Motor vehicle traffic as an air pollution exposure assessment method in big cities. Rev Bras Epidemiol 2011;14:120–30. http://www.scielo.br/scielo.php?script=sci_arttext&pid=S1415-790X2011000100011&lng=pt&tlng=pt

[28] Shairsingh KK , Jeong CH, Wang JM et al Characterizing the spatial variability of local and background concentration signals for air pollution at the neighbourhood scale. Atmos Environ 2018;183:57–68. 10.1016/j.atmosenv.2018.04.010

[29] Harris MH , Gold DR, Rifas-Shiman SL et al Prenatal and childhood traffic-related air pollution exposure and childhood executive function and behavior. Neurotoxicol Teratol 2016;57:60–70. 10.1016/j.ntt.2016.06.008 27350569 PMC5056808

[30] Open Street Map [Internet]. 2023. https://www.openstreetmap.org/

[31] Área Metropolitana de Lisboa. Plano metropolitano de adaptação às alterações climáticas. Volume I—Definição do cenário base de adaptação para a AML [Internet]. 2018. https://www.aml.pt/susProjects/susWebBackOffice/uploadFiles/wt1wwpgf_aml_sus_pt_site/componentPdf/SUS5BD0A09029884/PMAAC_AML_P021_VOL1_CENARIO_BASE_ADAPTACAO

[32] Nemery B , Hoet PHM, Nemmar A. The Meuse Valley fog of 1930: an air pollution disaster. Lancet 2001;357:704–8. 11247570 10.1016/S0140-6736(00)04135-0

[33] ESRI. ArcGIS: weighted overlay [Internet]. 2023. https://desktop.arcgis.com/en/arcmap/latest/tools/spatial-analyst-toolbox/weighted-overlay.htm

[34] Saaty RW. The analytic hierarchy process—what it is and how it is used. Math Model 1987;9:161–76.

[35] Paraskevis N , Roumpos C, Stathopoulos N et al Spatial analysis and evaluation of a coal deposit by coupling AHP & GIS techniques. Int J Min Sci Technol 2019;29:943–53.

[36] Sarkar S , Singh P, Lingala MAL et al Malaria risk map for India based on climate, ecology and geographical modelling. Geospat Health 2019;14:281–92. 10.4081/gh.2019.767 31724378

[37] Brender JD , Maantay JA, Chakraborty J. Residential proximity to environmental hazards and adverse health outcomes. Am J Public Health 2011;101:S37–52. 22028451 10.2105/AJPH.2011.300183 PMC3222489

[38] Malczewski J , Liu X. Local ordered weighted averaging in GIS-based multicriteria analysis. Ann GIS 2014;20:117–29. 10.1080/19475683.2014.904439

