# Supplementary material for: Mapping susceptibility to air pollution and its association with birth defects: a tool for public health intervention
Source: Eur J Public Health. 2025 Jun 11;35(5):947–53. doi: 10.1093/eurpub/ckaf077 (PMC12529273; doi:10.1093/eurpub/ckaf077)
Supplement: ckaf077_Supplementary_Data [file ckaf077_supplementary_data.zip › ckaf077_Supplementary_Data/ejph-2024-07-om-0506-File006.docx]

**Supplementary tables**

Table S1. Target population of the study, per year and total

| **Total sample** | | | | **Study area sample** | | | |
| --- | --- | --- | --- | --- | --- | --- | --- |
| **Years** | **Cases** | **Controls** | **Total** | **Years** | **Cases** | **Controls** | **Total** |
| 2016 | 27 | 51 | 78 | 2016 | 21 | 38 | 59 |
| 2017 | 38 | 37 | 75 | 2017 | 30 | 31 | 61 |
| 2018 | 37 | 37 | 74 | 2018 | 27 | 32 | 59 |
| 2019 | 40 | 58 | 98 | 2019 | 34 | 53 | 87 |
| 2020 | 22 | 24 | 46 | 2020 | 21 | 20 | 41 |
| 2021 | 40 | 42 | 82 | 2021 | 31 | 35 | 66 |
| 2016-2021 | 204 | 249 | 453 | 2016-2021 | 164 | 209 | 373 |

Table S2. Metadata of the geographic and alphanumeric data used in the study

| **Information** | **Type** | **Description** | | **Source** | **Date** |
| --- | --- | --- | --- | --- | --- |
| Residence location of cases and controls. | Alphanumeric information | | Residence location of mothers during pregnancy, based on the 7-digit postal code. | Barreiro Montijo Hospital Center | 2016-2021 |
| Postal code | Alphanumeric information | | Seven-digit postal code and its corresponding geographic coordinate | [https://www.codigo-postal.pt](https://www.codigo-postal.pt/) | 2021 |
| PRTR Activities^[[1]](#footnote-1)^ | Vector geographic information | | Activities under the national PRTR protocol | Agência Portuguesa do Ambiente | 2021 |
| Land Use Map (COS 2018) | Vector geographic information | | Land use type | Direção Geral do Território | 2018 |
| Roadways | Vector geographic information | | Main roadways considering the traffic volume | Open Street Map | 2021 |
| Elevation | Vector geographic information | | Contour lines and elevation points | Instituto Geográfico do Exército | 2009 |
| Administrative boundaries | Vector geographic information | | Municipal and parish boundaries | Direção Geral do Território | 2021 |

Table S3. Classification parameters of the factors of emission and concentration of air pollutants to the AP susceptibility map calculation.

| **Factor** | **Weight (%)** | **Category or range** | **Class** | **Degree of susceptibility** |
| --- | --- | --- | --- | --- |
| Distance to PRTR activities (m) | 37 | <2000 | 3 | High |
|  |  | 2000-4000 | 2 | Moderate |
|  |  | >6000 | 1 | Low |
| Density of PRTR activities per km² | 37 | >0,2539 | 3 | High |
|  |  | 0,2539-0,001 | 2 | Moderate |
|  |  | 0 | 1 | Low |
| Land use type | 13 | Industrial areas, infrastructures, landfills/dumps | 3 | High |
|  |  | Residential, commercial, recreational and tourism areas | 2 | Moderate |
|  |  | Natural, forest and agricultural areas | 1 | Low |
| Distance to major roadways (m) | 9 | <100 | 3 | High |
|  |  | 100-300 | 2 | Moderate |
|  |  | >300 | 1 | Low |
| Areas conducive to radiation fog formation | 4 | <17,5 | 3 | High |
|  |  | 17,5-40 | 2 | Moderate |
|  |  | >40 | 1 | Low |

1. Economic activities that report to the Pollutant Emissions and Transfers Registration protocol, which includes those that emit pollutants into the atmosphere, water and soil or transfer hazardous and non-hazardous waste outside the site, listed in national regulamentation [↑](#footnote-ref-1)
