# Supplementary material for: Mapping susceptibility to air pollution and its association with birth defects: a tool for public health intervention
Source: Eur J Public Health. 2025 Jun 11;35(5):947–53. doi: 10.1093/eurpub/ckaf077 (PMC12529273; doi:10.1093/eurpub/ckaf077)
Supplement: ckaf077_Supplementary_Data [file ckaf077_supplementary_data.zip › ckaf077_Supplementary_Data/ejph-2024-07-om-0506-File007.docx]

**Supplementary figures**

**
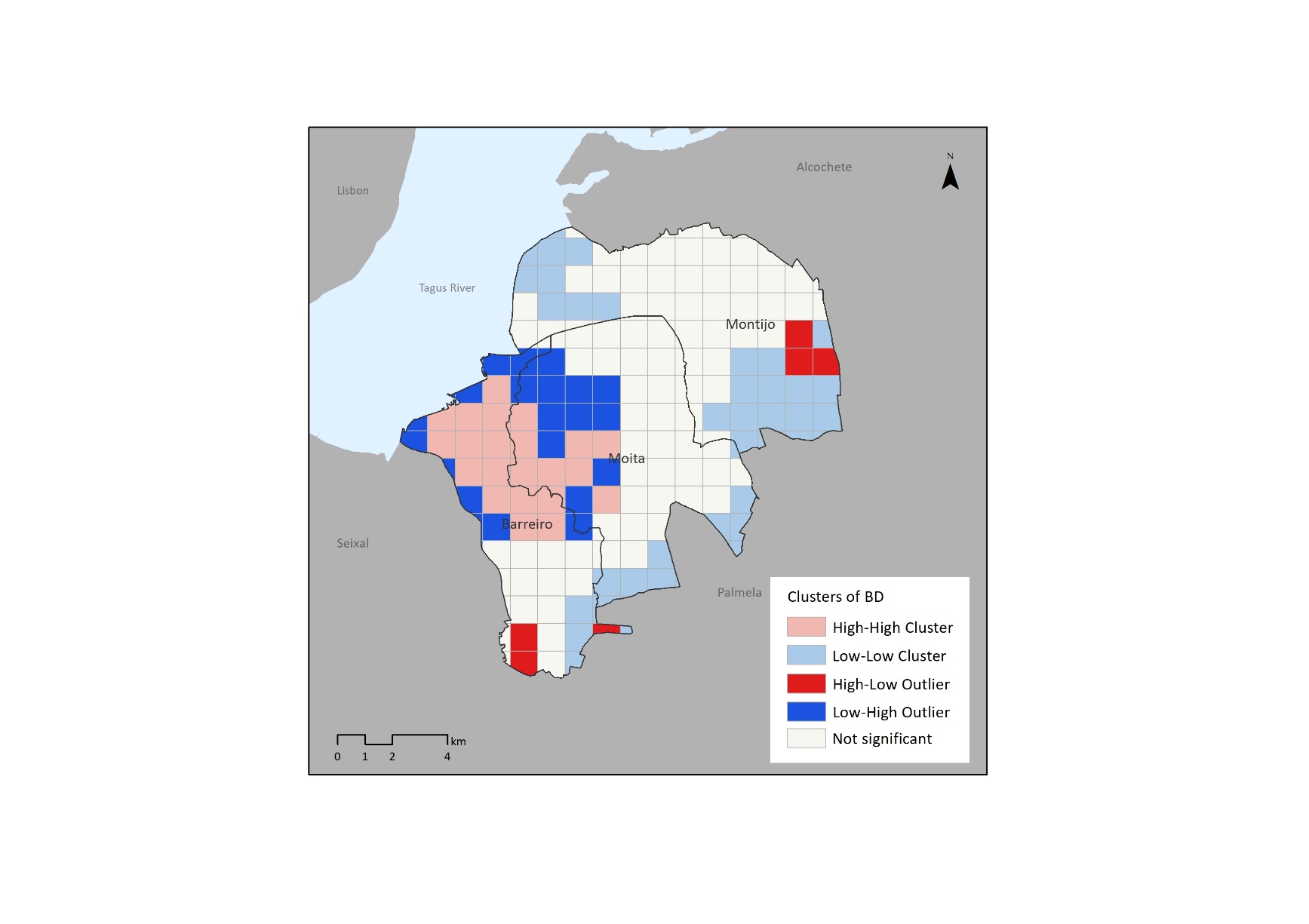
**


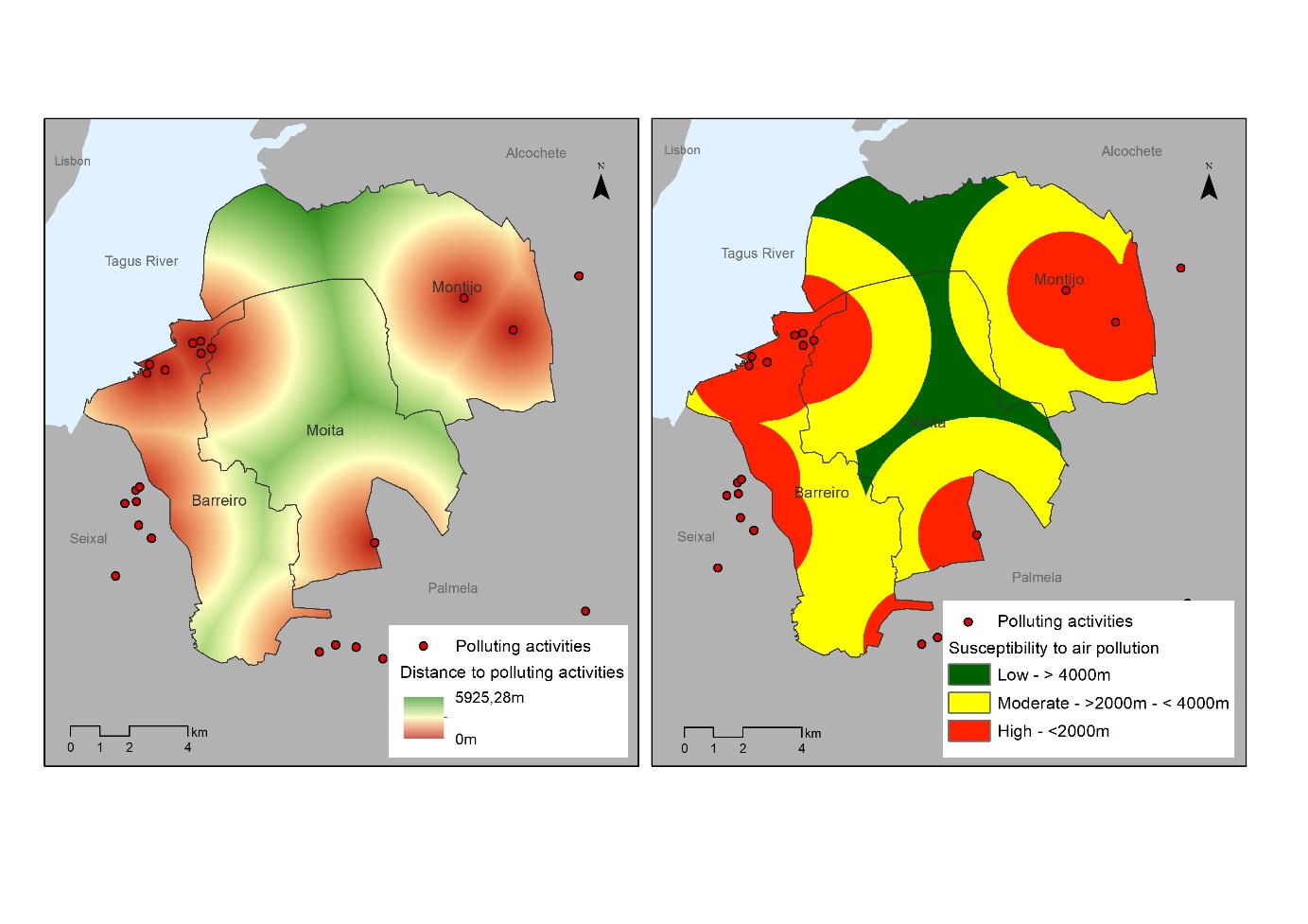
Figure S1. Cluster analysis in the municipalities of Barreiro, Moita and Montijo, according to the residence of the mothers of cases and controls, recruited for the study between 2016 and 2021.

Figure S2. Mapping of Euclidean distance to PRTR activities and its corresponding reclassification according to the defined susceptibility classes, for the residential areas of mothers of cases and controls recruited for the study between 2016 and 2021.


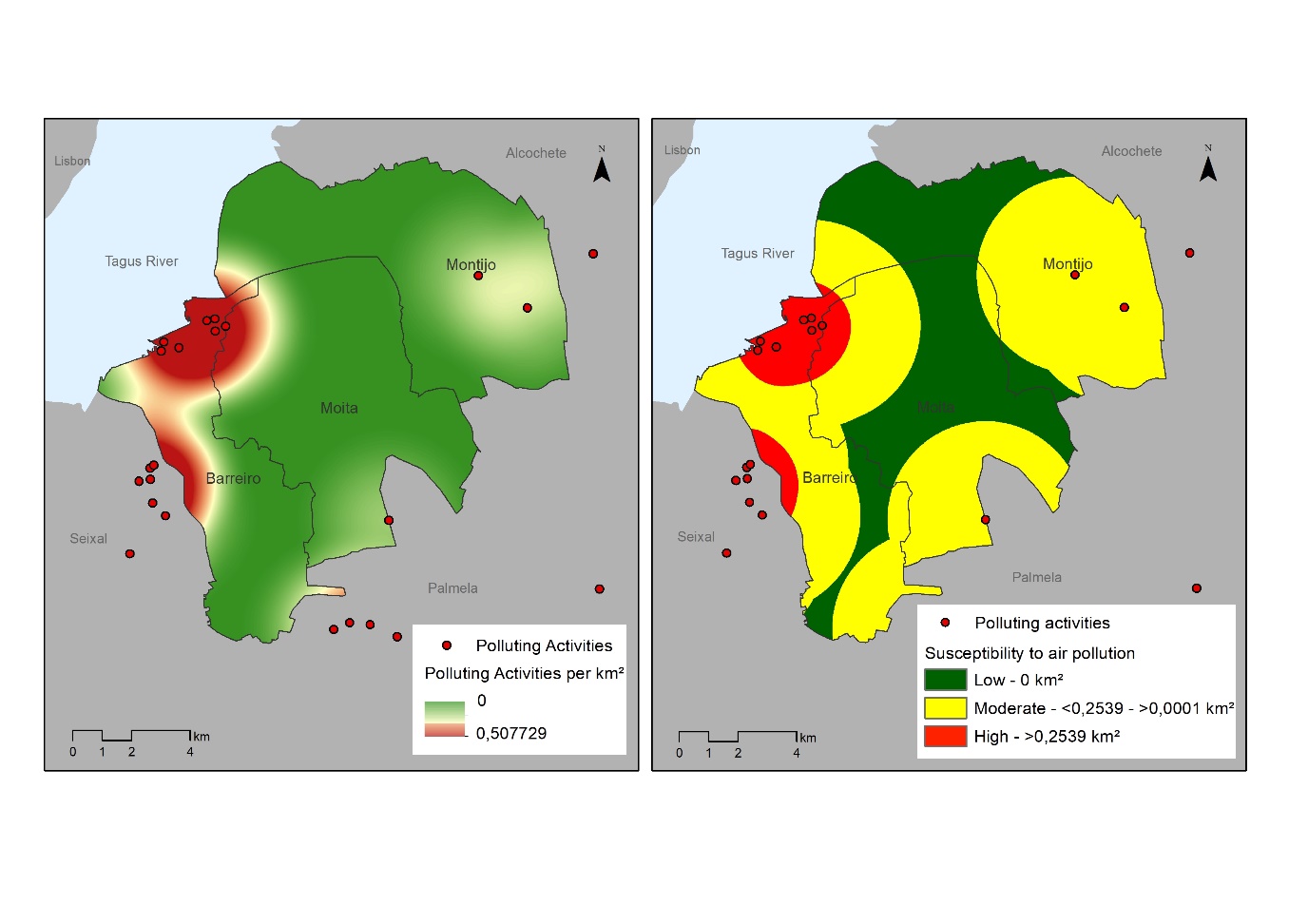
Figure S3. Mapping of the density of PRTR activities per km² and respective reclassification, according to the defined susceptibility classes, for the residential areas of mothers of cases and controls recruited for the study between 2016 and 2021.


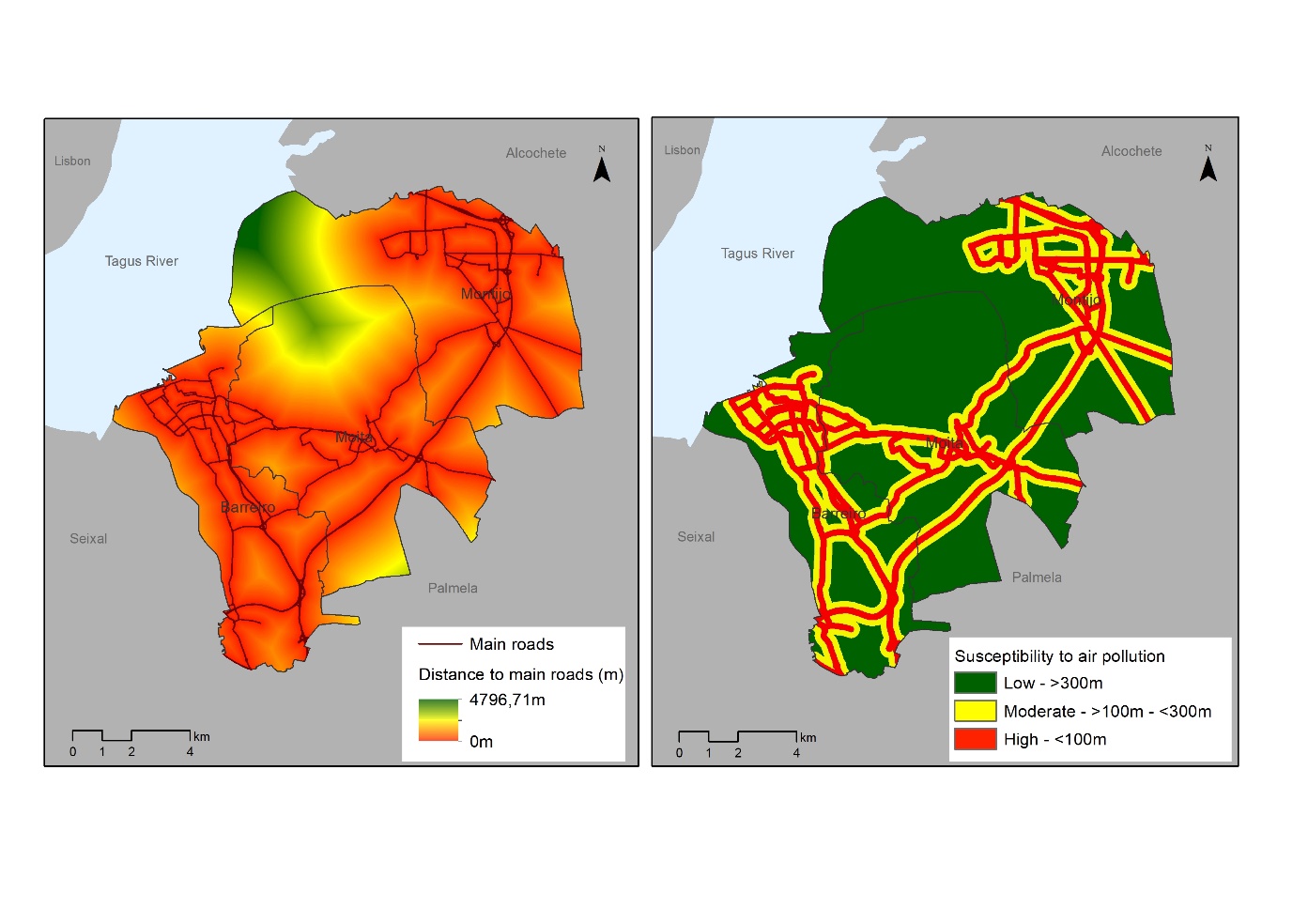


Figure S4. Mapping of Euclidean distance to major roadways and its corresponding reclassification according to the defined susceptibility classes, for the residential areas of mothers of cases and controls recruited for the study between 2016 and 2021.


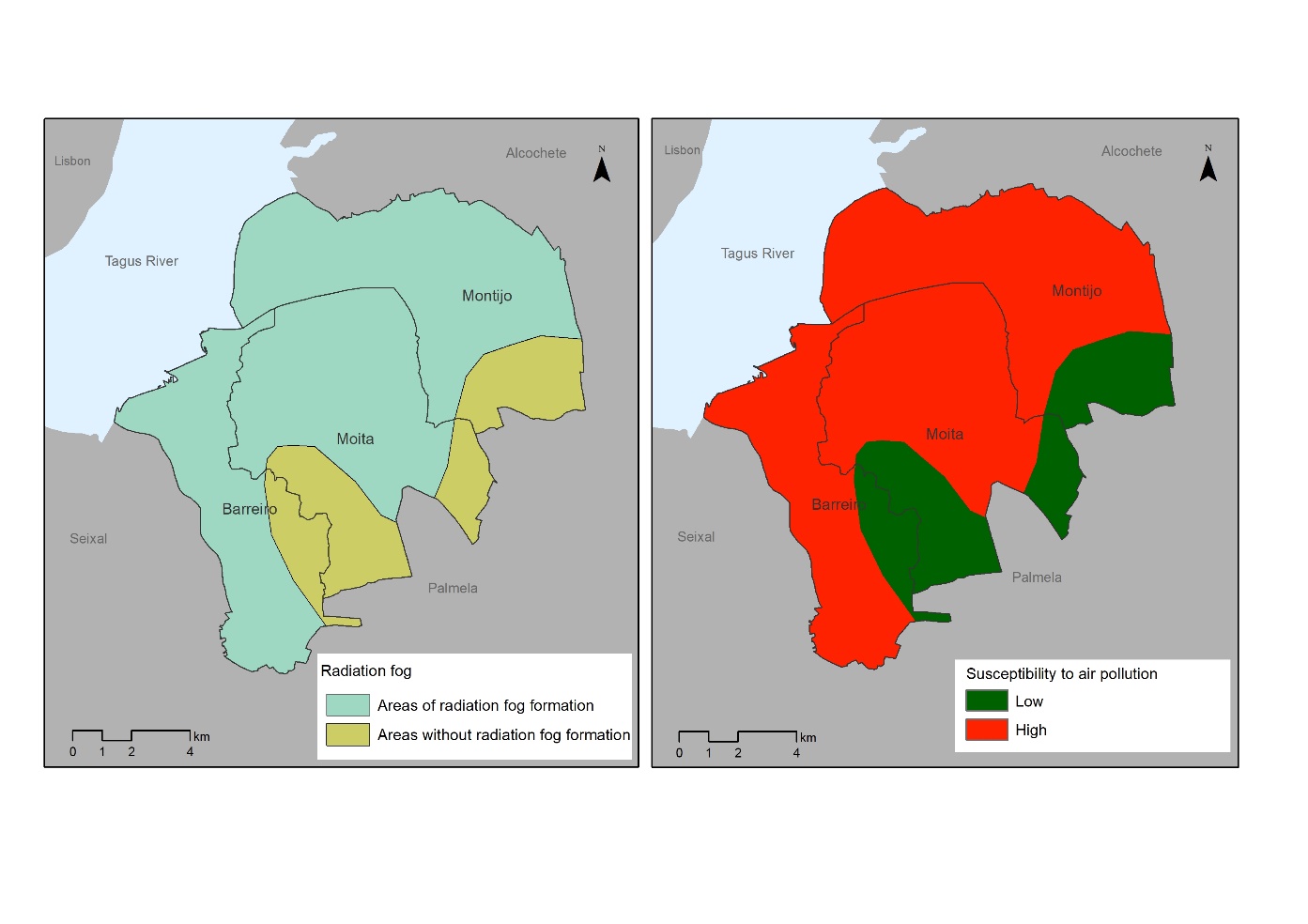

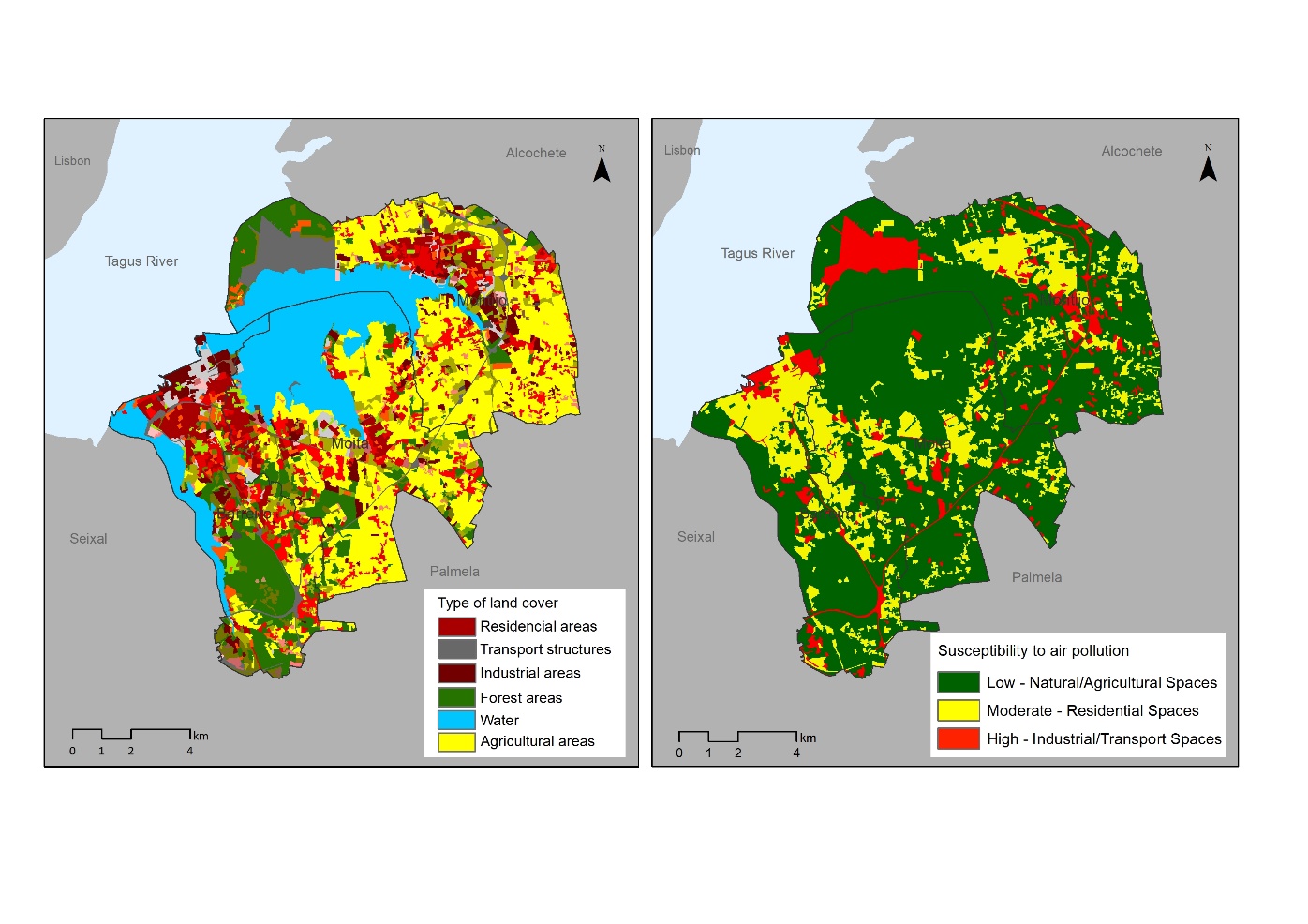
Figure S5. Mapping of the type of land occupation and respective reclassification, according to the defined susceptibility classes, for the residential areas of the mothers of cases and controls, recruited for the study between 2016 and 2021.

Figure S6. Mapping of Areas conducive to radiation fog formation and its corresponding reclassification, according to the defined susceptibility levels, for the residential areas of mothers of cases and controls recruited for the study between 2016 and 2021.


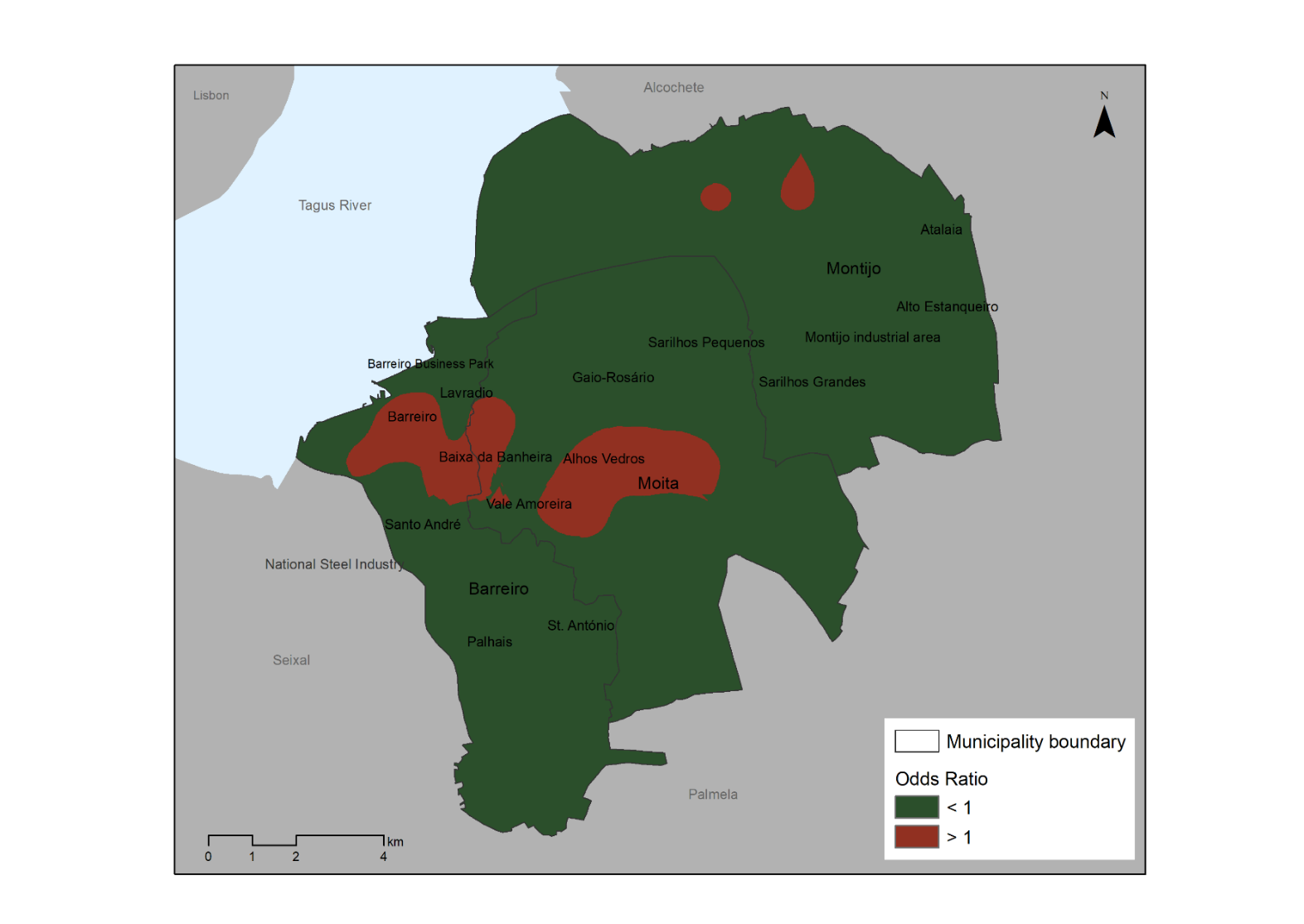
Figure S7. Map of the odds ratio for exposed and unexposed cases and controls, according to the residential areas of mothers recruited for the study between 2016 and 2021.
